# A Value Sensitive Scenario Planning Method for Adaptation to Uncertain Future Sea Level Rise

**DOI:** 10.1007/s11948-021-00347-0

**Published:** 2021-11-17

**Authors:** Anna Wedin, Per Wikman–Svahn

**Affiliations:** Division of Philosophy, KTH Royal Institute of Technology, Stockholm, Sweden

**Keywords:** Value sensitive design, VSD, Future scenarios, Scenario planning, Uncertainty, Climate change, Sea level rise, Adaptation, Ethics

## Abstract

Value sensitive design (VSD) aims at creating better technology based on social and ethical values. However, VSD has not been applied to long-term and uncertain future developments, such as societal planning for climate change. This paper describes a new method that combines elements from VSD with scenario planning. The method was developed for and applied to a case study of adaptation to sea level rise (SLR) in southern Sweden in a series of workshops. The participants of the workshops found that the method provided a framework for discussing long-term planning, enabled identification of essential values, challenged established planning practices, helped find creative solutions, and served as a reminder that we do not know what will happen in the future. Finally, we reflect on the limitations of the method and suggest further research on how it can be improved for value sensitive design of adaptation measures to manage uncertain future sea level rise.

## Introduction

Value sensitive design (VSD) was developed to address ethical values in the design of technological artifacts and systems (Friedman et al., 2003, 2021a). While initially developed for information technologies, it has since been applied to various technologies (Friedman & Hendry, 2019; Winkler & Spiekermann, 2018). A challenge in designing artifacts, systems or policies is that design decisions made today can have implications far into the future and that uncertain developments can impact the original design. This problem becomes critical when designing for long-term challenges, such as climate change.

This study combines VSD with scenario planning to study values involved in adaptation to the long-term challenge of sea level rise (SLR) caused by climate change. Global mean sea levels are rising and will likely continue to rise beyond this century (Oppenheimer et al., 2019). Adaptation to SLR is ultimately an ethical concern, raising questions relating to justice, responsibility, and values (Doorn et al., 2021; Thaler et al., 2018; Wedin, 2021). However, planning for adaptation to SLR is complicated as there is uncertainty of how fast and how high sea levels will rise, as well as uncertainty about how society will develop. Scenarios are standardly used to present the uncertainties of future climate change and SLR (e.g., Nicholls et al., 2014; Ranger et al., 2013; Valkering et al., 2010). Therefore, scenario-based approaches are natural starting points to investigate the value-dimension of adaptation to SLR.

This paper describes how VSD can be combined with scenario planning to study the value-dimension of planning for SLR in a case study concerning southern Sweden. VSD has not previously been used for climate change adaptation applications or urban planning for SLR, and the present study contributes to methodological development in both VSD and scenario planning. Therefore, it may be of interest for researchers in these areas and researchers interested in ethical issues in adaptation to climate change.

The paper is structured as follows: Sect. 2 reviews the literature on how uncertain futures have been addressed previously by VSD-based approaches. In Sect. 3, the proposed method, Value Sensitive Scenario Planning, is presented. Section 4 presents the case study in which we applied the method, as well as the results. In Sect. 5, we reflect on the results of the case study and the suitability of combining VSD and scenario planning and the potential to use this approach to study ethical issues in adaptation to SLR. Finally, Sect. 6 concludes with a discussion of limitations of the method and suggestions for further research and development.

## Value Sensitive Design for Uncertain Futures?

VSD departs from the idea that “all technologies to some degree reflect, and reciprocally affect, human values” (Friedman & Hendry, 2019, p. 1). The goal of VSD has been characterized as twofold: to support critical analysis of existing technology and to inscribe desired values into the design of new technology (Simon, 2016). The interaction between people and technology can enhance different values, and at the same time, technology can demote values unless care is taken that this does not happen. Our ambition in this study is to investigate ways to inscribe values into policies and measures for adaptation to SLR.

While VSD was initially developed for information technologies, it has recently been applied to large-scale infrastructure technological systems, including wind parks (Oosterlaken, 2015), biofuels (Palmeros Parada et al., 2018), and fossil gas production (Mouter et al., 2018). However, in these studies, as is the case for most studies based on VSD, an uncertain future and its implications on design are not explicitly addressed. One exception is Reuver et al. (2020), who address uncertainty in VSD applied to the problem how people will interact with a product. However, Reuver et al. do not explicitly discuss the future in which the product exists might develop nor how it might impact the analysis. Furthermore, the timescales in most cases of application of VSD are relatively short, concerning the next few years or decades. An exception is Yoo et al. (2016), who used VSD to envisioning future information systems for transitional justice in Rwanda, to enhance participants’ understanding of longer timeframes by placing their lifespan within a 200-year timeframe and in relation to different societal and technological developments. In doing this Yoo et al. guide “participants to effectively project themselves long into the future in their design thinking” (ibid, p. 4423). However, Yoo et al. do not explicitly address uncertainties nor how future societal development can affect the suitability of the design. De Wildt et al. (2019) identify value conflicts in smart electricity grids based on a literature review and point out that innovations can create new conflicts, suggesting a need for a more dynamic approach for ethics in technology.

Problems related to uncertainty about the future is also discussed in the wider literature of ethics of technology and engineering (e.g., Van de Poel & Royakkers, 2011, Chapter 8; Taebi, 2021, Chapter 2). The inherent uncertainty and risks of technology in society is a central topic in the scholarship of Sven Ove Hansson (e.g. 2013, 2017). Also, Ibo Van de Poel has emphasized the ethical implications of uncertainty of new technology, and argued that technologies should be seen as “social experiments.” (Van de Poel, 2016). Moreover, Philip Brey (2012, 2017) suggests that “anticipatory methods,” combining foresight analysis and ethical analysis to assess emerging technologies, should be developed. However, few concrete methods of this kind have been proposed so far.

In conclusion, we see a need for VSD-based approaches to more explicitly address extended time frames and uncertainty. The present study aims to contribute to the literature by linking VSD to methods from another field that focused on uncertainties-scenario planning. In the next section, we present our contribution to the literature by showing how VSD can be combined with scenario planning to study future long-term challenges.^1^

## A Value Sensitive Scenario Planning Method

Scenario planning offers a framework for addressing an uncertain future. Thinking about possible outcomes has probably always been done by humans to some extent, especially in the context of military tactics and strategy. The theory and practice of modern scenario planning are commonly set to the 1960s, with Herman Kahn’s work on scenario-planning in the Cold War context, followed by a broader use in society catalyzed by the oil crises in the 1970s (Dreborg, 2004; Spaniol, 2017). Commercial companies made subsequent developments, especially Royal Dutch Shell and General Electric (Bradfield et al., 2006). In recent years, environmental challenges, especially climate change, have prompted much development in scenario-based methods (EEA, 2009).

In scenario planning, a scenario is a description of a future, which is possible and also often required to be plausible (Maier et al., 2016). Moreover, the purpose of scenarios is usually very specific: to inform decision-making (EEA, 2009). Finally, more than one scenario is often used, thus creating a set of scenarios that describe multiple plausible futures (Maier et al., 2016). Börjesson et al. (2006) devise a typology of three different types of scenarios: predictive, explorative, and normative scenarios. In this typology, the scenarios in this study are *explorative scenarios* because they “explore situations or developments that are regarded as possible to happen” (ibid. p. 727). Börjesson et al. (2006) also note that scenarios can either describe an end-state (at a particular time in the future) or a process (a development path into the future). The scenarios we have developed in this study describe a process and serve a very specific purpose: as a tool to elicit values in planning for uncertainty in future sea level rise (as described in Sect. 4 below).

**Fig. 1:**
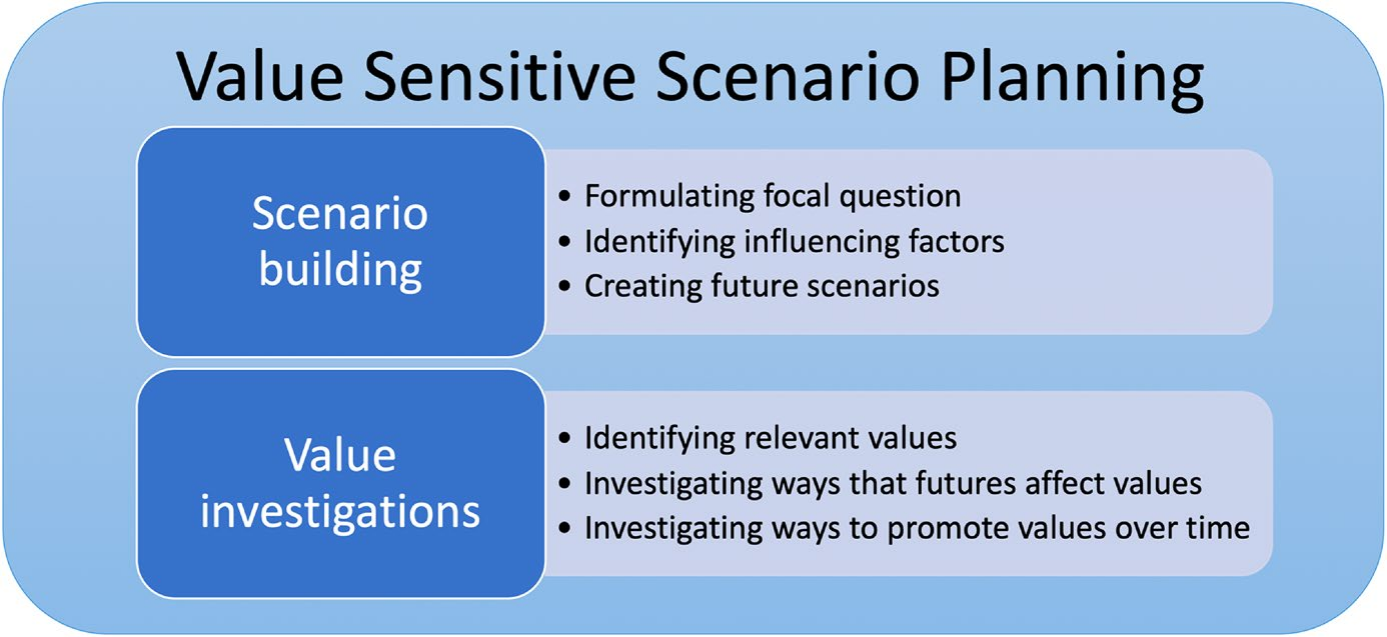
Elements of the method Value Sensitive Scenario Planning

Standard scenario planning, however, does not address ethical issues or give any guidance concerning how to approach questions of values and value conflicts; for this, we turned to VSD. Concretely, our method consists of a combination of two parts: (1) building scenarios and (2) investigating value-implications of these scenarios. See Fig. 1.

The scenario-building part consists of the development of tailor-made scenarios for the case study. The methodological inspiration for this part is based on Carlsen et al. (2013), which in turn is based on the “intuitive logic approach” (van der Heijden, 2005). A vital principle of the method devised by Carlsen et al. (2013) is to involve the users of the scenarios in the development of the scenarios. Following this principle, the process starts by defining a “focal question” informed by the purpose of the study. Scenarios are then constructed in a workshop setting. The focal question and the scenarios we developed in our case study are described in the next section.

An essential methodological choice we made in this study was to not include values directly in the scenarios. The reason was that we wanted to study the influence of different future developments (e.g., regarding the sea level, political and economic development) on adaptation for SLR and the value-related implications of different adaptation options in these different futures. Therefore, in our assessment, we assumed that society’s values in the future would be sufficiently similar to the present that our analysis would be helpful. This assumption is, of course, a simplification, as values might change over time. Because we cannot know for certain how values will evolve in the future we are faced with an additional type of uncertainty, which Taebi et al. (2020) call “evolutionary normative uncertainties.” Hence, our approach does not address such evolutionary normative uncertainties. However, it seems reasonable to assume that more fundamental values might not change very much. For example, it seems plausible that happiness will continue to be deemed positive in the future and that suffering will be something negative. Finally, by not building values into the scenarios, the analysis becomes more straightforward and more transparent. In future developments of our approach, value change could perhaps also be incorporated, for example, in line with the proposal by van de Poel (2018).

The second part of the method consists of value investigations in the context of uncertain future scenarios. VSD has been characterized as “tripartite investigations” – conceptual, empirical, and technological investigations (Friedman & Hendry, 2019). Conceptual investigations identify direct and indirect stakeholders and identify and define the values and potential value conflicts implicated by a technology. Empirical investigations “examine stakeholders’ understandings, contexts, and experiences in relation to technologies and impacted values” (Davis & Nathan, 2015, p. 16). Finally, technological investigations assess how the technology impacts values. Friedman and Hendry (2019) state that all three parts should ideally be used in an iterative and integrative manner. However, a review by Winkler and Spiekermann (2018) found that almost no existing VSD studies engage with all three parts.

The value investigation in our method consists of three steps that build on each other, corresponding to the three steps of the tripartite investigations of VSD. The first step aims to identify values at stake and can be seen as a conceptual investigation.^2^ More specifically, we use a bottom-up approach to value elicitation, in which the stakeholders (in our case: representatives for the stakeholders) identity values.^3^ The bottom-up approach is opposed to using a setlist of pre-defined values (Umbrello & van de Poel, 2021), and is thought to lead to increased levels of ownership of the results among the workshop participants. Note that this step could be carried out at the beginning of the method before beginning with scenario building. In the second step of the value investigation, values are discussed in the context of the different scenarios. Concretely, we did this in a workshop setting (described in the next section). The participants were asked how the different scenarios influence how different values are likely to be promoted or realized. In the tripartite investigation of VSD, this corresponds to an empirical investigation, as it investigates how stakeholders believe that values can be promoted in a given surrounding (Friedman & Hendry, 2019). The third step of the value investigation is also the final step of our method and is inspired by the technological investigation of the tripartite VSD-methodology. This step has a more proactive approach in aiming to find a system solution for a specific problem. The next section describes the method in more detail by explaining the steps in case study.

## The Method Applied to a Case Study of Adaptation to SLR in Southern Sweden

Coastal towns and cities all over the world will need to adapt to rising mean sea levels (Oppenheimer et al., 2019). Adaptation to SLR can take a wide variety of different forms, for example avoiding building in areas prone to future flooding, constructing barriers, using nature-based protection, or using waterproof materials for building houses. Theoretically, the fundamental goal of adaptation can be seen to protect that which we value (Hartzell-Nichols, 2011). Therefore, in choosing among adaptation options, values and ethical concerns are critical (Baard et al., 2012).

A central challenge for societies that need to adapt to SLR is that changes in sea levels will play out over centuries, even if global greenhouse gas emissions were to completely stop today (Zickfeld et al., 2017). This is a problem because the uncertainty in SLR over longer timescales is substantial. Projections of global mean SLR range from ca 0.2 to over 2 m by the year 2100 (Garner et al., 2018; Sweet et al., 2017). After the year 2100, the uncertainty for SLR becomes even more severe. It is difficult to quantify this uncertainty in terms of probabilities, as large sources of uncertainty are due to technological, economic, and political developments and how these will impact the future concentrations of greenhouse gases in the atmosphere. Moreover, these developments will also be impacted by people’s choices in the future (e.g., climate policies). Planning for situations of this kind has been called “decision-making under deep uncertainty” (Hansson & Hirsch Hadorn, 2016; Marchau et al., 2019). Lempert et al. (2003) define deep uncertainty as “the condition in which analysts do not know or the parties to a decision cannot agree upon (1) the appropriate models to describe interactions among a system’s variables, (2) the probability distributions to represent uncertainty about key parameters in the models, and/or (3) how to value the desirability of alternative outcomes.” (pp 3–4). The magnitude and speed of global mean SLR over the next centuries is clearly a situation of deep uncertainty (van der Pol & Hinkel, 2019).

Moreover, adaptation is not only challenged by uncertainty regarding SLR but also by various socioeconomic developments that affect adaptive capacities. This is another type of deep uncertainty because it is extremely difficult to predict social, economic, political, and regulatory changes and how these might impact the constraints and opportunities of adaptation options. For example, compare the U.S. climate and environmental policies under President Obama to those under President Trump.

Because adaptation to SLR is at its core a local issue, it has to be studied in a context. The magnitude of local SLR differs from the global mean, depending on regional oceanological and geological factors. Our case study, which was carried out within a research project focused on ethical adaptation to SLR, consisted of municipal adaptation to SLR in southern Sweden. Southern Sweden is more affected by SLR than the northern parts of the country, in which SLR is partially counteracted by the postglacial land uplift. This means that the global mean SLR scenarios will have almost full impact on southern Sweden (Hieronymus & Kalén, 2020). In Sweden, coastal municipalities already battle coastal erosion, and the problems will become more severe as sea levels are rising (Storbjörk & Hedrén, 2011). Adaptation to SLR is a primary concern for the built environment and is, therefore, an issue for urban and regional planners who are responsible for the design and implementation of adaptation policy.

We implemented the method in a series of workshops with municipal and regional planners from southern Sweden. The participants in the workshops were recruited to be part of the research project through our contacts and cannot, therefore, be seen as a random selection of stakeholders. However, we believe that the participants in the case study have a good understanding of the interests of a wider group of stakeholders. Henceforth, when we speak of “we,” we mean us researchers and authors of this paper, and when we speak of “participants,” we mean the participants of this part of the research project (see "Appendix 1" for a list of the affiliations of the participants).

The first step at the first workshop was to formulate a focal question together with the participants: “What factors might be most impactful for a Swedish coastal municipality in managing rising sea levels in a 200-year time frame?” Notably, the focal question used a 200-year time frame, far longer than typical planning horizons in municipal planning in Sweden (von Oelreich et al., 2013). Admittedly, we had already told the participants that we wanted to examine longer timeframes in our research. However, participants were free to choose a focal question, including a shorter timeframe, but the participants still wanted to use the 200-year time horizon. The focal question was then used in a “structured brainstorming” session (Carlsen et al., 2013), resulting in categories of different factors. The second step of our method consisted of identifying influencing factors. After the first workshop, the results were synthesized into thirteen external factors and used to construct a “morphological field” (Carlsen et al., 2013). The resulting morphological field consisted of two factors related to the speed and knowledge of SLR and nine factors related to the socio-economic and political development in the municipality and Sweden (see "Appendix 2" for the morphological field).

Building on the iterative element of VSD, we briefly paused from the scenario building and the second workshop started with a session devoted to values. The participants were given an exercise that used “envisioning cards,” which have been developed as a VSD method for exploring questions regarding values, stakeholders, time, and pervasiveness (Friedman & Hendry, 2019). After this, we returned to scenario building together with the participants, following the approach of Carlsen et al. (2013). Our work with scenario building continued through the third workshop, by the end of which we had constructed five scenarios). The scenarios were developed into text narratives by us before the fourth workshop. Before the workshop, we solicited feedback from the participants on the scenario narratives (see "Appendix 3" for the resulting text narratives of the scenarios).

In workshops four and five, we used the scenarios to examine the following questions:

1. What values are essential for adaptation to SLR in this context?
2. How are these values affected in different future scenarios?
3. Which adaptive measures are best suited, given society’s values in different scenarios?

In small groups, the participants identified several values, including both moral and non-moral values and instrumental and intrinsic values. The values identified by the participants ranged from more abstract values such as justice to more concrete values like functioning sewerage. After having identified relevant values, the groups gathered to share and discuss their findings. Next, each group was given two out of the five scenarios to discuss how society’s ability to promote essential values would be affected in the respective scenarios.

The results from the workshops included a list of values that the participants thought were important (see "Appendix 4"). We have chosen not to focus on the results in this article because the paper's focus is on the methodology rather than the exact values. However, here follows some results of conflicts between values that we found to be interesting. First, the participants identified a value conflict between economic and environmental values, where the former often is prioritized. They suspected that this conflict would be enhanced if resources end up being scarce in the future. Counteracting this, however, might lead to conflicts between justice and individual freedom. A second result was that the speed of SLR was thought likely to affect motivation to act, especially at the local level. Unless the change is dramatic, there is a risk that significant decisions will be postponed. Clear guidelines and regulations from the government level were identified as essential to help avoid this. A third result was that the participants noted that strong regulations might lead to conflicts between the collective good and individual freedom.

Finally, the groups were asked to turn to the third question, which concerned how society should adapt to protect these values. Since the participants work in different municipalities, we chose to contextualize the questions by asking the participants to have a “typical” southern Swedish municipality in mind rather than thinking of an actual, specific municipality. The reason was to prevent discussions from getting stuck at factual details and make the results more generally applicable to different contexts. As such, the discussion was expected to be more general than a detailed technological investigation of a specific location or adaptation measure. The results here were not as clear cut; as the participants pointed out, it seems pretty likely that several adaptation measures would be needed in the different scenarios, but at different points in time. For scenarios with greater SLR, hard protection like walls were thought to be insufficient, and managed retreat was deemed an unavoidable solution. The focus shifted towards what the individual could do in scenarios with limited resources or limited state action. In addition to these scenario-specific adaptation measures, several general aspects that need to be accounted for in adaptation policy were lifted. For this session, too, a concluding discussion followed the work in small groups.

After the final workshop, we compiled the results from the different groups in order to improve the overview and ease further analysis. In the next and final section, we reflect on the method in light of the results and the case study, and point to some strengths and areas of improvements.

## Benefits of the Method

This section identifies five main benefits or contributions of this method based on the results of an evaluative survey to the participants. First, the participants found that the method provided a framework for discussion of planning for the future. The participants appreciated a space to discuss ethical aspects of adaptation in unusually long time-perspectives. Second, the method enabled the identification and articulation of essential values. For example, one participant said that the method clarified that what we consider to be of value can influence which adaptive measures are chosen. A third benefit was that the method was perceived as helping to challenge established planning practices. Today, public policy in Sweden is often guided by economic models, where many of the values identified in our study are not included. For example, economic cost–benefit analyses were questioned by some participants. Working with scenarios contributed to further insights on how we can improve adaptation practices. One participant wrote that they had realized that different future scenarios give different points of departure for adaptation options. Another participant wrote that the scenarios brought up many examples and details previously not considered, which can be helpful as a foundation for the identification of considerations that need to be accounted for in adaptation policy.

A fourth benefit was that the method helped to find creative solutions. When planning far into the future, creativity and imagination are necessary. One understanding of design as a process is that it consists of both rationality and creativity (Franssen et al., 2018). Rationality is concerned with choosing between actions based on specific criteria, whereas creativity generates these options. Our method strengthens the rational dimension of design by providing a clear methodology for deliberation, while at the same time promoting creativity by broadening horizons and thus generating new options and ideas.

The fifth and final benefit we identified on the basis of the responses of the participants was that the method served as a reminder that we do not know what will happen in the future. Amongst municipal and regional planners in Sweden, there seems to be a desire to predict what will happen and plan for that specifically, especially when planning for SLR (Carlsson Kanyama et al., 2019). One participant said that while the scenarios felt distant from reality, it was good that potential development over unusually long time-horizons were discussed, since “after all, we do not even know what will happen within our lifetime.” This result is in line with the purpose of scenario planning to widen the perspectives and be more prepared for the uncertain future.

Now that we have listed the significant benefits we found with the approach, we also need to discuss the remaining problems and avenues for further research, which is the focus of the next and final section.

## Limitations and Future Research

The method combined elements from VSD with elements from scenario planning, which were successful in that it enabled a discussion on values that were found useful by the participants. The case-study also resulted in values and value-conflicts that we will use in continued work in studying ethical issues in long-term ethical adaptation to SLR. Hopefully, both the method and the case study can be of interest for researchers in other contexts, for example for ethical issues in adaptation to climate change. Our study suggests that VSD can provide values in new domains, strengthening the case for VSD, a point also made by Friedman et al. (2021b). At the same time, our findings suggest a need for further development. This section highlights limitations and aspects that could be the topic of future research.

First, we note that our study only engaged with parts of VSD. While we covered all three parts in the tripartite investigation in VSD, the emphasis was on conceptual analysis (value elicitation, identifying potential value conflicts). Moreover, fuller stakeholder identification and engagement would have been more ideal. Because we relied heavily on values identified by our participants, which consisted of a small group of people, we likely missed other important values. Barnett et al. (2016) argue that “[v]alues are ‘situated’ in specific social contexts in that they are influenced by people’s experiences and daily practices, and the places and cultures in which these are embedded.” A more comprehensive stakeholder engagement would therefore likely strengthen the social acceptability of adaptation and ethical acceptability (van der Kaa et al., 2020).

Second, our study would have benefitted from a process extending over a longer time. For example, one participant said that more time would be necessary to make the method truly useful in an actual situation. In theory, it would be possible to make a more comprehensive study by evaluating how those living and working in a studied municipality perceive their coastal environment and how they engage with the identified values, thus deepening the empirical investigation. Also, a more realistic and direct policy-informing objective would have been better. For example, it would be interesting to examine a specific adaptation measure in a specific context, together with norms and design requirements that would follow the identified values. One could follow suggestions by van de Poel et al. (2013) on how to transform values into concrete design requirements. To continue working in this way would be recommended as VSD is meant to be an iterative process.

Third, we see a need to improve the value investigation part of our method. The results show a range of values that need to be considered in adaptation. However, when a diversity of values needs to be adhered to, this can lead to a paralysis of adaptation action or lead to contradictory outcomes (Adger et al., 2008). Finding the best practices for the initial discovery of values has been discussed in a recent overview of eight grand challenges of VSD (Friedman et al., 2021b). As mentioned in the previous section, we believe that a fuller engagement with the identified values to draw out and highlight the most important ethical values would have strengthened our work. A simple method for achieving this consists of repeatedly asking “why?” when a value is proposed, eliciting underlying values (Friedman et al., 2006). However, such an engagement would increase the risk of us interpreting the results and verifying that which we already know. We are aware that this risk is also present in our study, and were this method to be used for an actual planning decision, this would have to be addressed.

A fourth limitation is that while our method helps identify values of importance to adaptation and potential value conflicts, it does not provide any answers to how we should prioritize or deliberate in such situations. One criticism facing VSD is that the realization of values is left undetermined (Manders-Huit, 2010). It has been argued that VSD needs to commit to an ethical theory to provide any accurate guidance (e.g., Jacobs & Huldtgren, 2018). Therefore, it would be valuable to link our method to a specific theory or principle for adaptation to guide ethical deliberation. For example, Van de Poel (2015) proposed different approaches for dealing with conflicting values, and van de Kaa et al. (2020) presents an application of some of these to the case of smart metering. It would be interesting to explore these approaches in connection to our method.

Finally, there are particular problems related to looking far into the future. Much has happened in the last 200 years, and there will be significant changes in the coming centuries. As has already been discussed in Sect. 2, it could be the case that the values identified today will not be considered necessary in the future or that other values may become more critical. For example, de Wildt et al. (2019) suggest that an “evolutionary account of morality” should be considered essential in the ethics of technology. We decided not to include values directly in future scenarios, which should be addressed in future studies that more explicitly considers evolutionary normative uncertainties (Taebi et al., 2020), for example, in the line of the proposals of van de Poel (2018). Another issue related to long-term changes is that it is difficult to imagine the possibilities that might open up in the future. For example, not many people in the 1820s would have anticipated a future in which everybody is connected via invisible strings, enabling access to vast sources of information at a low cost, especially as the telegraph was not even invented at the time! In the same vein, in 200 years, it is likely that society will be technologically advanced in ways that are difficult to imagine. In the light of this, a critic might argue that it is a waste of time engaging in scenarios for such extreme periods. However, our method is not about predicting the future or finding answers for what should be done in the year 2100 or year 2200. Instead, the purpose is to start thinking about the long-term consequences of decisions that need to be made in the near term. Other approaches for decisions under deep uncertainty have similar purposes (Marcahu et al., 2019). For example, the “Dynamic Adaptive Policy Pathways” (DAPP) approach (Haasnoot et al., 2013) is a method to construct dynamic or “flexible” strategies to manage uncertain futures. Evaluating different policy options in different future contexts, emphasizing values, seems a potentially helpful complement to DAPP and similar approaches.

To conclude, while there is room for improvement, our method presents a framework for including ethical values in the long-term planning under uncertainty, for the design of technology and policy. Instead of resigning to biases towards the status quo, we believe that engaging in scenario planning and adding a value element can help make society more open to potential changes, and thus better prepared to meet an uncertain future.

## Appendix 1 Workshop details

**Table:**

Workshop	Date	Location	Focus of workshop	Output
1	2018–10-16	Trelleborg	Scenario building	Focal question Morphological field
2	2019–10-21 – 2019–10-22	Halmstad	Scenario building	n/a
3	2020–02-06	Malmö	Scenario building	Five scenarios
4	2020–06-08	Online (Zoom)	Value investigation	List of relevant values
5	2020–06-17	Online (Zoom)	Value investigation	Notes from discussions

**Table:**

Participants representing	
Swedish geotechnical institute SGI	
Båstad municipality	
Halmstad municipality	
Laholm municipality	
Mörbylånga municipality	
Trelleborg municipality	
Ystad municipality	
Halland county administrative board	
Skåne county administrative board	
The foundation Halland county museums	
National Board of Housing, Building and Planning	
Swedish transport administration	
Swedish national heritage board	
Swedish Agency for Marine and Water Management	

## Appendix 2 Morphological field

**Table:**

Global sea level rise	Certainty of prognosis of future sea level rise	Priority of sea level rise in municipality	Municipal economic development	State grants for adaptation	State intervention	Availability of material and resources	Availability of innovation and technology	Economic inequality	Population development	Responsibility
0.3 m year 2100, 0.4 m year 2200	Less certainty	Very high priority	Negative growth	Large	More state intervention	Good	Good	More than today	Steady	Insignificant changes compared to today
0.5 m year 2100, 0.95 m year 2200	Same as today	High priority	Steady growth	Medium	Similar as today	Limited	Limited	Same as today	Rapid increase	Insurance companies and banks get more demanding
1.0 m year 2100, 2.8 m year 2200	More certainty	Medium priority	High growth	Low	Less state intervention	Poor	Poor	Less than today	Depopulation	Property owners get more responsibility
1.5 m year 2100, 5.1 m year 2200		Low priority	Large economic fluctuations	None						Property owners get less responsibility
2.0 m year 2100, 7.5 m year 2200		Very low priority	New economic system							
2.5 m year 2100, 9.7 m year 2200										

## Appendix 3 Scenarios

### Scenario A

The overarching consensus that had been defining the global climate collaboration at the beginning of the century was replaced by protectionism and short-term measures to boost the global economy after the Pandemic 2020–2021. Emissions from fossil fuels continued to rise as the price for oil and coal fell rapidly after the chaos that characterized the global economy and political landscape after the Pandemic. In the 2030s, it became evident that the collapse of the West Antarctica had begun and progressed faster that had been feared. The swift changes in Antarctica and the substantial body of research showing that a large and fast sea level rise was to be expected led to the formation of a UN organ dedicated to sea level rise: UNSEAL (United Nations Sea-Level Organization). Subsequently, decisionmakers were presented with more accurate predictions for the development of sea level rise and were thus able to plan and allocate resources for the large investments that were needed to handle sea level rise in coastal towns and cities around the world. Sea levels rose slowly at first but accelerated after a while. The global mean sea level was 0.44 m higher year 2050, 1.5 m year 2100, 3 m year 2150 and a whole 5 m higher year 2200 (compared to year 2000).

From the 2030s, the international emphasis on sea level rise led to the EU and the Swedish government putting aside more resources for coastal municipalities to handle floods, protect critical infrastructure, and relocate important services. State funds, however, were not sufficient for the large measures the municipalities needed to protect existing residential areas and infrastructure. Most municipalities in southern Sweden realized that they needed to increase tax revenues to afford the large investments necessary to protect against the sea, but this proved difficult, due to the poor economic development and the widespread unemployment in Sweden and the EU.

The inability for the municipalities to handle sea level rise led the central government to pass laws and guidelines which controlled and regulated domains that the municipalities previously had been in charge of. Insurance companies and banks were the first to realize the threat of sea level rise, which manifested in properties in flood risk areas no longer were able to be insured or mortgaged. Quick changes in property prices in locations near the sea, especially in the cities, was a contributing factor for recurrent and large fluctuations in the Swedish economy.

The fictional municipality in southern Sweden needs to put a lot of effort and resources into trying to handle the effects of the rapid sea level rise, as other municipalities in Sweden and globally has experienced. The municipality has had a fast population development, due to immigration from southern Europe, increased number of births per capita, and people living longer.

### Scenario B

The 2020s became the turning point when it was no longer possible to ignore the effects of climate change, in part because of wild fires and storms that grew in frequency and magnitude. Another contributing factor for the global awakening was humanity’s common experience of the waves of pandemics that spread during the same decade. The global community went through a life crisis but emerged at the other end, unified in the belief that human rights should be at the top of the agenda and that the economic system should transform into a circular economy with an emphasis on redistribution. A sustainable political system with significantly lower levels of emissions broke the neoliberal political trend which had been dominating globally until the 2020s.

In this new world order, a lot of effort was put into understanding climate change and limit its negative consequences. This increased focus on climate change and sea level rise put its mark on science, and huge research efforts led to better predictions for the future development of sea level rise. This in turn enabled better planning and allocation of resources for the large measures that needed to be done in coastal towns and cities globally. Despite significant reductions in emissions it turned out to be difficult to stop the ongoing changes in the earth’s climate system. Feedback mechanisms caused vast amounts of greenhouse gas to be released from the melting permafrost in the arctic region and from the many large wildfires around the globe. Climate change was particularly large at the poles, and the fast melting of the glaciers at Greenland during 2020–2050 was followed by the collapse of West Antarctica by the end of the century. The sea rose to 0.34 m year 2050, 1.0 m year 2100, 1.8 m year 2150 and 2.8 m year 2200 (compared to 2000).

The rapid sea level rise made it all the more common with extreme water levels that lead to huge societal costs and caused much attention in Sweden in the 2040s. Some residents in coastal areas decided to move, but many stayed in the municipality. In Sweden too, there was a shift to a circular economy and less economic inequality. The Swedish administrative model with a decentralized rule and municipal planning monopoly has persevered. Even though the individual property owner’s responsibility had crystalized further and insurance companies had become stricter in the sense that insurance premiums for properties in coastal zones had been raised significantly, there are central state funds available for climate adaptation, both for individual property owners and for municipalities.

In this scenario, the fictional municipality in southern Sweden, like most other municipalities in Sweden and globally, puts much emphasis on handling the fast sea level rise. There are good resources in terms of material for building barriers to the sea, as well as advanced technology and innovation. The population has a steady increase similar to the early 2000s.

### Scenario C

In the 2030s, the world experienced several summers with extreme draught, which combined with more wildfires put a lot of pressure upon the agricultural sector and food production. This led to an increased competition of land, which in turn led to growing tensions and political instability globally. Increased economic inequality resulted in uneven economic development. At the same time, emissions from fossil fuels escalated and global warming continued, becoming visible especially at the poles. The political unrest and poor economic development globally made climate change into one problem among many more urgent problems that needed to be solved. Sea level rise thus was also not a prioritized topic within the research community, which meant that little progress was made in understanding the processes driving sea level rise, and the uncertainty of prognosis therefore was even greater than at the beginning of the millennia. The fast melting of the glaciers at Greenland during 2020–2050 was followed by the collapse of West Antarctica by the end of the century. The sea rose to 0.34 m year 2050, 1.0 m year 2100, 1.8 m year 2150 and 2.8 m year 2200 (compared to 2000).

Even though sea level rise was noticeable for municipalities in southern Sweden during the 2040s, the issue was not given much attention due to other more urgent problems. This was the case even though recurring floods brought significant costs onto financially already pressured organizations. The Swedish administrative model with a decentralized rule and municipal planning monopoly has persevered and the legislation regarding responsibility for adaptation is unchanged. The state funds and support that were requested at the beginning of the twenty-first century has not showed up and the municipalities and property owners have to do what they can to face sea level rise.

Our fictional coastal municipality struggle with negative economic growth for large parts of the twenty-second century, as well as with a steady emigration from the municipality. The economic inequality is great. There are neither economic nor technical resources to protect important areas against flooding. The situation is feeling increasingly hopeless for those living near the sea, as the insurance companies even are unwilling to ensure properties that are frequently flooded.

### Scenario D

The recurring crises caused by the extremely rapid sea level rise dominated the twenty-first century. Towards the end of the 2020s it was evident that the collapse of Antarctica had begun and that it happened much faster than had been thought possible. Sea level rise thus became one of the most important global political issues and in the 2030s the UN organ UNSLOW (United Nations Sea-Level Organization Worldwide) was founded. UNSLOW were given the mission to coordinate international efforts to stop the collapse of the glaciers at Antarctica and Greenland and turned to largescale geo-engineering. Unfortunately, these efforts proved unsuccessful, and were terminated. The changes in Antarctica shifted attention and resources towards research on the topic, causing the body of knowledge to grow quickly. As a result, it was possible already by 2050 to predict with high certainty that sea levels would rise by over two meters by 2100, and that the inertia of the system would lead to an even higher sea level rise in the following century. The scientific understanding of where we were heading was very clear, even though many people long refused to accept the prognoses. Sea levels rose by 0.63 m by year 2050, 2.5 m year 2100, 5 m year 2150, and 10 m year 2200!

The municipalities in southern Sweden began to notice the sea level rise already during the 2030s. The first signs were extreme water levels, which had been quite rare in the twentieth century, causing major floods in several municipalities. The floods meant huge economic costs leading municipalities to shift the attention to the problem of flood risk and sea level rise. The municipalities did not receive much funding or guidelines from the EU or the Swedish state, but due to a positive economic development in the municipality, there were sufficient resources for long term planning and adaptation to constant sea level rise. Despite this, the municipality did not act. Property owners were the ones who had to pay for the protection of their houses and when insurance companies stopped insuring low lying properties, the consequence was that many houses were abandoned and teared down.

The rapid sea level rise made it difficult to get enough sand for beach nourishment. The international interest in protecting communities against the sea led to the development and implementation of new technology. This includes Nano-technology that can be used to cover existing buildings, and smart barriers which can be installed and autonomously rise when needed. Methods for moving houses were developed and increasingly applied. The fictional municipality in southern Sweden needs to put a lot of effort and resources into trying to handle the effects of the rapid sea level rise, as other municipalities in Sweden and globally do too. The municipality has had a fast population development, due to immigration from southern Europe, increased number of births per capita, and people living longer.

### Scenario E

The trends of 2000–2020, with increased globalization and economic and technological development continued in a similar manner. The pandemic 2020 was not followed by any significant political and economic changes. A rapid increase of renewable energy sources and electric transports finally made emissions go down in the 2030s, and through extensive use of carbon capture technologies, the world finally saw decreasing levels of carbon dioxide by 2100. The glaciers on Greenland and Antarctica continued melting, but it did not happen as quickly as had been feared. Even though the sea levels rose somewhat, there was a hope that if global temperatures went down somewhat, global mean sea levels would stabilize. In such a way, future options are limited and the uncertainty of the prognosis can be said to have lessened significantly. In 2100, the sea was only 0.5 m higher than it was in 2000 and by 2200 it had risen by 1 m.

Since sea level rise with relatively high certainty will not be that dramatic, adaptation to it has a fairly low priority in the fictional municipality. Focus is rather on continued development of activities to shift society further in. the direction towards negative emissions. This is also the focus for the Swedish state, but there are also some grants available for climate adaptation.

In our municipality, there are limited material resources for adaptation, due to the limitations on emissions that exists nationally. In terms of innovation and technology, resources are also limited as expertise has mostly been developed in other fields. The Swedish administrative model with a decentralized rule and municipal planning monopoly has persevered and the legislation regarding responsibility for adaptation is unchanged. The economic development, naturally with a few shorter exceptions, has been steady and in line with the Swedish average during the twenty-first and twenty-second centuries, and the economic equality is more or less unchanged compared to 2020.

## Appendix 4 List of identified values

The environment.

Natural beauty.

Environmental sustainability.

Natural habitats.

Social sustainability.

Justice.

Accessibility.

Equality.

Equity.

Integration.

Liberty.

Freedom.

Democracy.

Safety.

Quality of life.

Health.

Recreation.

Pedagogical value.

Sense of place.

Rural culture.

Economic sustainability.

Industry.

Tourism.

Availability to sites.

Infrastructure.

Transport.

Water and sewerage.

## Footnotes

1 Note that the types of scenarios and methods in scenario planning and described in the following section, differ significantly from the use of “value scenarios” in VSD, which is about creating narratives and envisioning the way in which a user interacts with a system or product rather than planning for the future (Friedman and Hendry 2019).

2 In the VSD literature, stakeholder identification is also put forward as a central part of conceptual investigations. In our case, since we were working with planners with a good understanding of who the direct and indirect stakeholders in adaptation to SLR are, we focused on values instead.

3 In this study, we adhere to the broad VSD definition of a value as that which a person or a group of people finds important, with a special focus on social and ethical values (Friedman and Hendry 2019). Important values do not only include things that we already have that need to be protected, but also aspects which adaptation can create an opportunity to enhance.
